# A unique approach for a large intra-thoracic traumatic tracheo-oesophageal fistula: A case report from Syria^☆^

**DOI:** 10.1016/j.ijscr.2021.106087

**Published:** 2021-06-11

**Authors:** Bassam Darwish, Amjad Sikaria, Ameer Kakaje

**Affiliations:** a Department of Thoracic Surgery, Al Mouwasat University Hospital, Damascus University, Damascus, Syria; b Faculty of Medicine, Damascus University, Damascus, Syria; c University Hospital Geelong, Barwon Health, Victoria, Australia

**Keywords:** Intercostal muscle flap, Fistula, Trauma, Tracheo-oesophageal, Oesophageal leak

## Abstract

**Introduction and importance:**

Acquired tracheo-oesophageal fistula (TOF) is a rare complication of intubation or traumas, either blunt or penetrating. In a penetrating chest trauma, the closure of TOF can be challenging and requires a unique technique. A flap can and intra-tracheal tube can also be used. We present this case to demonstrate a unique late presentation of TOF and the unique approach that was used.

**Case presentation:**

A patient presented with a large TOF caused by shrapnel, and was surgically managed after two months of the injury by using a smaller intra-tracheal tube, and using an oesophageal wall flap to close the tracheal defect and intercostal muscle flap was used for the oesophageal wall repair. The postoperative intrathoracic oesophageal leak was successfully treated conservatively.

**Clinical discussion:**

Although the surgery could not be conducted until 2 months after the injury, the approach used was successful and the patient was able to resume his normal life after the surgery. The flap from the oesophagus and intercostal muscles and using a smaller tracheal tube successfully repaired the TOF with minimum stress on the suterings, and the conservative approach for the leak was also successful.

**Conclusion:**

Traumatic TOF management can be complicated, but we speculate that using a smaller tube with the conservative management of the complications was ideal for the TOF acquired from a shrapnel.

## Highlights

• Acquired tracheo-oesophageal fistula (TOF) can be caused by trauma.
• The closure of TOF can be difficult and requires unique technique.
• Using a smaller intra- tracheal tube with a flap from oesophageal wall can help in TOF closure in the tracheas.
• Oesophagus wall injury from the shrapnel was managed by intercostal muscles.
• Post-operative oesophageal leak can be managed conservatively.

## 1 Introduction

Tracheo-oesophageal fistula (TOF) is when the wall of trachea or bronchi becomes irregularly connected with the oesophagus. This defect can be congenital or acquired and has a negative impact on the patient and quality of life as it causes dysphagia, aspiration pneumonia and weight loss [1]. Traumatic tracheo-oesophageal fistula (TOF) is a rare complication of a penetrating trauma, and is hard to be diagnosed by physicians. Large TOFs impose a greater challenge and require sophisticated techniques. We present a large TOF caused by a penetrating trauma that was surgically managed by a unique technique by using a smaller size of intra-tracheal tube, intercostal muscles and a partial wall of the oesophagus to close the TOF. This work has been reported in line with the SCARE 2020 criteria [2].

## 2 Case report

A 41-year-old man suffered from recurrent coughing episodes from recurrent aspiration for six weeks when eating or drinking. These symptoms started after a shrapnel injury from a mortal shell penetrated the right third intercostal space from the back, medially to the shoulder. The patient denied any significant personal or family history. The patient lived with his wife and children and had no significant psychological or genetic history. He also lost around 8% of the weight due to unsatisfactory nutritional status. Corrective surgery to remove the shrapnel could not be performed at that time due to the conflict around the city, and therefore the surgery was conducted around 2 months from the injury (2 weeks after presenting to the hospital).

**Fig. 1:**
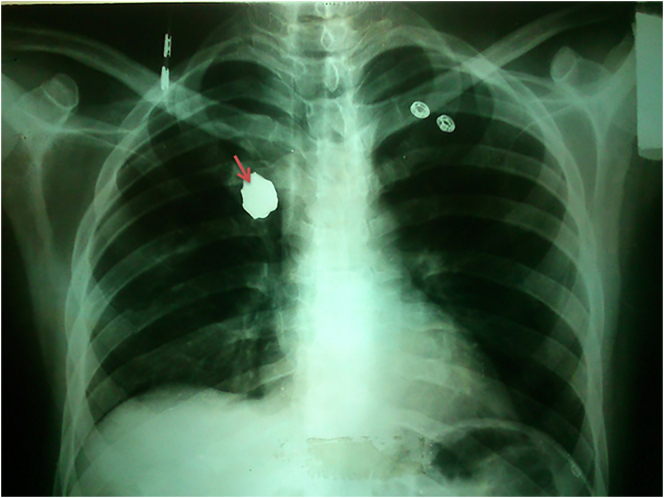
Showing the CXR with the shrapnel in the right hemithorax (red arrows).

**Fig. 2:**
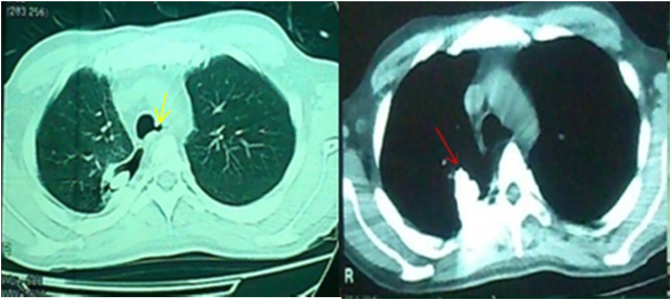
Showing CT-scan of the thorax with the shrapnel (red arrow) and fistula (yellow arrow). The shrapnel was at the postero-internal portion of the right hemithorax near the mediastinum at 4th thoracic vertebrae level.

Chest x-ray (CXR) showed the shrapnel in the right hemithorax near the mediastinum (Fig. 1). Computed tomography (CT) scan (Fig. 2) showed the shrapnel placed in the postero-internal portion of the right hemithorax near the mediastinum at the 4th thoracic vertebrae level. Endoscopy proved the opening of the fistula in the oesophagus. Bronchoscopy found a 4 cm fistula opening, 2 cm superiorly to the carina of the trachea. The fistula involved the tracheal membrane wall and extended 2 cm to the left bronchus. The fistula was partially covered by granulomatous tissue and massive thick mucus secretions. There was no stenosis detected. Furthermore, a bronchoalveolar lavage (BAL) was taken for culture which was normal.

**Fig. 3:**
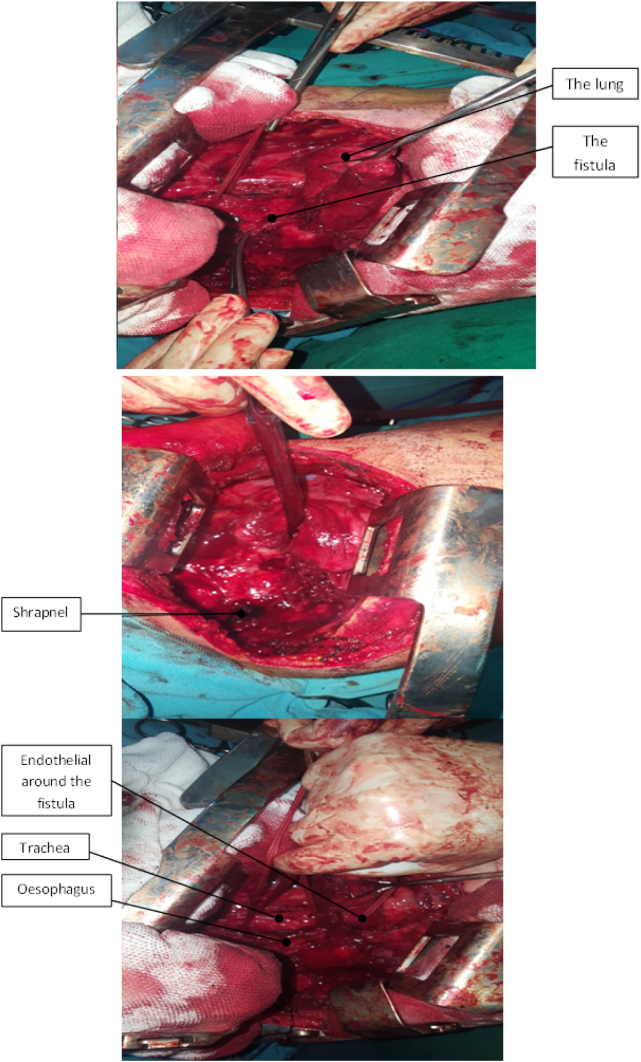
Showing the fistula in the surgery.

Trans-thoracic surgical approach was indicated (Fig. 3) and the surgery was conducted in Al Mouwasat University Hospital by the leading author and the second author. A small (size 35) tracheal tube that has double-lumen was inserted into the left bronchus and the right lung was isolated. A right posterolateral thoracotomy in the 4th intercostal space was performed. Multiple adhesions were released between the lung and the pleura. The shrapnel in the postero-internal portion of the pleural cavity was found and removed. Then, the fistula and the defect in the posterior wall of the trachea and left bronchus and in the anterior wall of the oesophagus measuring 4 cm in length were found. This defect measured 2 cm at the trachea and carina, and it included 2 cm of the left main bronchus. To get over this difficulty, the tracheal and left bronchial defect were sutured without stretching while trying to avoid future stenosis by using a part of the oesophageal wall that was not damaged by the shrapnel; two flaps that were well-perfused were used from the oesophagus to cover the lesion in the trachea and each measured around 2 × 0.5 cm. The lesion in the wall of the oesophagus was also longitudinally sutured around the naso-gastric tube (35 Fr.), which was a smaller size than the normal size (normal size is 39 Fr). Using the oesophageal wall and a smaller tube were believed that this will provide less stress when suturing and better outcomes. After the trachea and oesophagus were sutured, an intercostal muscle flap was placed between them to help with the oesophageal injury from the shrapnel. A chest drain tube was installed and the incision through the chest wall was sutured. Then, laparotomy was performed, and a jejunostomy catheter was inserted. There was no need for mechanical ventilation.

**Fig. 4:**
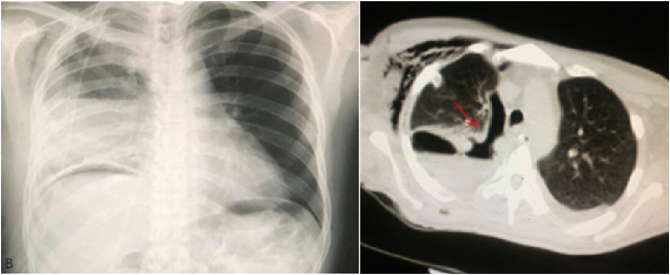
Which shows: the CXR on the left with the cavity and sub diaphragmatic free gas which was iatrogenic from laparotomy, and the CT-scan on the right with the cavity with air-fluid level (red arrows).

The patient remained NPO, and his condition was relatively well post-operatively. Nutrition was introduced by jejunostomy and the wife helped in management and was very cooperative. Daily CXRs were taken which found a right cavity with air-fluid level on the third day of surgery (Fig. 4), despite the chest tube being in place at that time. A chest CT scan was performed (Fig. 4) which confirmed the pleural cavity. Subsequently, the chest tube drainage was removed, and a CT-guided new drainage tube was installed into the pleural cavity. It drained 200 ml of saliva-like fluid and air. Methylene blue oral administration yielded blue dye from the drainage tube which indicated an oesophageal leak. A conservative approach was indicated and NPO remained for another 2 weeks. The patient was later permitted to drink water and other soft liquids as he could tolerate it despite the leaking oesophago-pleural-cutaneous fistula. Further CXRs were taken, and methylene blue administration was weekly repeated. The amount drained from the tube gradually decreased until it ceased approximately 24 days after the surgery, and chest tube was removed on the 31st days after the surgery.

The patient was told to come back to the hospital if any symptoms reoccurred as he now lives in a place accessible to the hospital. The patient recovered well and came back after four months for check-up, and he was well and started to regain weight and had a normal CXR.

## 3 Discussion

Trauma comes in third as a causative factor for TOFs. Chest wall crush injuries that occur in road-traffic accidents from the impact of steering wheel are the main reason for traumatic fistulas [3,4]. In penetrating chest trauma, the location of fistula is determined by the perforation site [5] and shrapnel track. Contrast radiography and using fiberoptic with endo- or bronchoscopy are the main methods of diagnosis for TOF. Furthermore, endo- or bronchoscopy can provide more details as they can determine the exact location and extension of TOF [5,6]. Traumatic TOF management usually follows guidelines which include patient assessment, the location of the fistula, detection and treatment of any respiratory infection, and patient's nutrition status. The patient in our case did not have significant respiratory infections and had a relatively good nutrition status. This work also adds to the literature the approach of using a smaller intratracheal tube when using an oesophageal flap in addition to the conservative approach of the leak.

Surgery is the treatment of choice, and the surgical technique depends on multiple factors such as fistula length, size, and tracheal stenosis and other comorbidities. The trans-thoracic approach is indicated when the TOF is immediately above the carina, or if there is a broncho-oesophageal fistula [7]. Posterolateral thoracotomy in such cases will be performed where unilateral left lung intubation will be used, and the incision will be made at the right fourth intercostal space level [7]. In order to successfully close the fistula, viable tissue between the two sutures such as a muscle flap from either from the neck or intercostal should be used [3,4,7,8].

We used a particular technique to facilitate the tracheobronchial suturing; we used a lower size of intra- tracheal tube (size 35 Fr) instead of (39 Fr.) and a partial section from the oesophageal wall to close the tracheobronchial defect. We did this by taking a small section from the oesophageal wall when performing the excision on fistula to use it to cover the damaged area of the membranous section of the posterior trachea. Afterwards, a muscular flap from intercostal muscles was inserted between the trachea and the oesophagus to help with the oesophageal injury. This was performed by taking out the area around the fistula opening in the oesophagus and stuttering it with the damaged area in the trachea which resulted in having a small section of the oesophagus in the posterior membranous trachea to compensate for the damaged area.

There have been reports on using latissimus dorsi muscle and pericardial flap instead of intercostal muscle flap [9,10]; as the surgeon believed a tight closure is required for the fistula, the intercostal muscles was used instead of latissimus dorsi which is believed to be better for wide areas but not good to cover a fistula tightly [9]. Furthermore, although primary repair with pericardial flaps was reported, they are unsafe as they are poorly vascularised and can paradoxically move, mainly with positive pressure ventilation [10].

This resulted in a good healing of the suture, but it may have caused stretching in the oesophageal wall which may result in leak or stenosis. No sign of stenosis occurred in the our case, and the leak was managed conservatively.

Postoperative extubation is preferred, and postoperative ventilation should be avoided [7,8]. Postoperative oral feeding should also be avoided after the surgery for 10–14 days because of the high risk of oesophageal leak [7]. Our case had a leak after surgery which was conservatively and successfully managed by installing a drainage tube in the newly formed pleural cavity after the leak and by ceasing oral feeding. Having financial hurdles and living in a remote area has caused the delayed and improper treatment of this patient until being transferred later on to the central hospital which is similar to multiple other cases in Syria [11], which is similar to our patient who lived in a conflict zone in an area distant to the main hospitals.

Ideally, the patient should be assessed after such a trauma for the psychological impact. However, this is rarely done in Syria due to limited resources and the stigma of mental health. More CT scans could not be taken due to limited resources as well and they were thought not to change the management as the patient became better after the surgery. Finally, the patient adherence and his cooperative family were believed to be key in helping the patient to become better.

We recommend this technique of using the oesophagus wall, intercostal muscles and a smaller intra-tracheal tube, as we believe it helped in covering the lesion with minimum tension when suturing and provided reliable flaps with good vascular supply and minimum complications that were managed conservatively. The smaller intratracheal tube was speculated to help to reduce the tension as well.

## 4 Conclusion

Acquired tracheo-oesophageal fistula (TOF) is a rare complication of a penetrating trauma. The closure of longer and larger defects can be performed by tracheobronchial suturing with a smaller size intra- tracheal tube and using a partial section from the oesophageal wall to close the tracheal defect which were believed to help in reducing the tension on the suturing. A postoperative oesophageal leak was conservatively managed.

## Ethical approval

These research ethical standards were reviewed and approved by Damascus University faculty of medicine.

## Source of funding

We received no funding.

## Author contribution

Bassam Darwish MD: Methodology; Visualization; Supervision; Validation; Investigation; original draft; Project administration.

Amjad Sikaria MD: Resources; Supervision; Investigation; Investigation; review.

Ameer Kakaje MD: Conceptualization; Formal analysis; Software; original draft; Writing - review & editing.

## Consent

Written informed consent was obtained from the patient for publication of this case report and accompanying images. A copy of the written consent is available for review by the Editor-in-Chief of this journal on request.

## Guarantor

Professor Bassam Darwish.

## Provenance and peer review

Not commissioned, externally peer-reviewed.

## Patient perspective

The patient was cooperative and happy by the procedures done as he regained weight and normal eating after the management.

## Declaration of competing interest

No conflict of interest to declare.

## References

[1] Bibas B.J. (2016). Surgical management of benign acquired tracheoesophageal fistulas: a ten-year experience. Ann. Thorac. Surg..

[2] Agha R.A., Franchi T., Sohrabi C., Mathew G., Kerwan A., Thoma A. (2020). The SCARE 2020 guideline: updating consensus Surgical CAse REport (SCARE) guidelines. Int. J. Surg..

[3] Couraud L., Ballester M.J., Delaisement C. (1996). Acquired tracheoesophageal fistula and its management. Semin. Thorac. Cardiovasc. Surg..

[4] Reed M.F., Mathisen D.J. (2003). Tracheoesophageal fistula. Chest Surg. Clin. N. Am..

[5] Bibas B.J. (2018). Surgery for intrathoracic tracheoesophageal and bronchoesophageal fistula. Ann. Transl. Med..

[6] Chauhan S.S., Long J.D. (2004). Management of tracheoesophageal fistulas in adults. Curr. Treat. Options Gastroenterol..

[7] Paraschiv M. (2014). Tracheoesophageal fistula—a complication of prolonged tracheal intubation. J. Med. Life.

[8] Diddee R., Shaw I.H. (2006). Acquired tracheo-oesophageal fistula in adults. Contin. Educ. Anaesth. Crit. Care Pain.

[9] Miyata K., Fukaya M., Nagino M. (2020). Repair of gastro-tracheobronchial fistula after esophagectomy for esophageal cancer using intercostal muscle and latissimus dorsi muscle flaps: a case report. Surg. Case Rep..

[10] Rafieian S., Asadi Gharabaghi M. (2018). Tracheopleural fistula after thoracoscopic esophagectomy: novel therapeutic approach with pericardial and intercostal muscle flaps. J. Surg. Case Rep..

[11] Ghareeb A., Kakaje A., Ghareeb A., Nahas M.A. (2020). An enormous arteriovenous malformation presenting in a child in sacro-gluteal region and managed successfully by recurrent embolisation and surgery. Int. J. Surg. Case Rep..

